# Adult Subependymal Neural Precursors, but Not Differentiated Cells, Undergo Rapid Cathodal Migration in the Presence of Direct Current Electric Fields

**DOI:** 10.1371/journal.pone.0023808

**Published:** 2011-08-31

**Authors:** Robart Babona-Pilipos, Ilia A. Droujinine, Milos R. Popovic, Cindi M. Morshead

**Affiliations:** 1 Institute for Biomaterials and Biomedical Engineering, University of Toronto, Toronto, Ontario, Canada; 2 Institute of Medical Science, University of Toronto, Toronto, Ontario, Canada; 3 Lyndhurst Centre, Toronto Rehabilitation Institute, Toronto, Ontario, Canada; 4 Department of Surgery, University of Toronto, Toronto, Ontario, Canada; 5 Department of Rehabilitation Science, University of Toronto, Toronto, Ontario, Canada; City of Hope National Medical Center and Beckman Research Institute, United States of America

## Abstract

**Background:**

The existence of neural stem and progenitor cells (together termed neural precursor cells) in the adult mammalian brain has sparked great interest in utilizing these cells for regenerative medicine strategies. Endogenous neural precursors within the adult forebrain subependyma can be activated following injury, resulting in their proliferation and migration toward lesion sites where they differentiate into neural cells. The administration of growth factors and immunomodulatory agents following injury augments this activation and has been shown to result in behavioural functional recovery following stroke.

**Methods and Findings:**

With the goal of enhancing neural precursor migration to facilitate the repair process we report that externally applied direct current electric fields induce rapid and directed cathodal migration of pure populations of undifferentiated adult subependyma-derived neural precursors. Using time-lapse imaging microscopy *in vitro* we performed an extensive single-cell kinematic analysis demonstrating that this galvanotactic phenomenon is a feature of undifferentiated precursors, and not differentiated phenotypes. Moreover, we have shown that the migratory response of the neural precursors is a direct effect of the electric field and not due to chemotactic gradients. We also identified that epidermal growth factor receptor (EGFR) signaling plays a role in the galvanotactic response as blocking EGFR significantly attenuates the migratory behaviour.

**Conclusions:**

These findings suggest direct current electric fields may be implemented in endogenous repair paradigms to promote migration and tissue repair following neurotrauma.

## Introduction

Neural precursor cells (NPCs) in the adult brain are promising candidates for the development of strategies to repair central nervous system (CNS) tissue following injury or disease [1]. Adult NPCs (comprising both stem and progenitor cells) reside in a well-defined region lining the lateral ventricles known as the subependyma (SE). Under baseline conditions, adult NPCs migrate to the olfactory bulb where they differentiate into mature neurons. Following injury several studies have demonstrated the migration of SE-derived NPCs to the site of injury, both in the presence or absence of exogenous factors that activate the cells [2]–[4]. Factors such as epidermal growth factor (EGF) [3], the cytokine stromal cell-derived factor-1α (SDF-1α) [5]–[6], and microglia derived inflammatory molecules [7] have been proposed as guidance cues that promote the migration of NPCs toward lesion sites. Post-injury NPC migration toward the site of injury has not been fully characterized and is relatively limited. The ability to enhance the migratory capacity and subsequent integration of NPCs into newly generated tissue would be beneficial for the development of neural repair strategies.

Physiological direct current electric fields (dcEFs) play important roles during development and in tissue repair [8]–[11] and have been shown to cathodally direct the turning of growth cones during axon elongation [12]–[13]. In the context of wound repair, keratinocytes and corneal epithelial cells have been shown to migrate cathodally in the presence of dcEFs similar in strength to those that arise at the site of skin and cornea lesions during wound healing [14]–[15]. The phenomenon by which cells migrate in a directed manner in the presence of an electric potential gradient is termed galvanotaxis. A previous study by Li et al. [16] demonstrated that a subpopulation of cells – primarily neuroblasts – within an explant of embryonic neural tissue undergoes cathode-directed galvanotaxis. A more recent study by Meng et al. showed that adult hippocampal cell line-derived NPCs (HCN-A94 cells) exhibit cathodal galvanotaxis in the presence of 250 mV/mm and 500 mV/mm dcEFs [17]. The existence of endogenous dcEFs in the mammalian brain [18] raises the possibility that wound-induced dcEFs (that may arise, for example, following stroke) may play a role in guiding endogenous NPCs to the site of injury.

Given our work demonstrating the significant contribution of endogenous SE-derived NPCs to tissue regeneration and functional recovery following stroke [3], we asked whether adult SE-derived NPCs could be induced to undergo cell body translocation in a rapid and directed fashion in the presence of a dcEF. Importantly, we examine the effects of dcEFs on differentiated neural cells as the ability to selectively target NPCs is an important consideration for developing neural repair strategies. Herein we have used live cell time-lapse imaging to perform an extensive kinematic analysis on pure populations of adult SE-derived NPCs and their differentiated progeny. We demonstrate rapid and directed cathodal migration of NPCs *in vitro* in the presence of a dcEF. The migration persists only for as long as the dcEF was applied, and removal of the dcEF results in the quick diminution of galvanotaxis. Moreover, we show that NPC cathodal galvanotaxis is unchanged in the presence of continuous media cross-perfusion demonstrating the phenomenon is a direct effect of the electric field and not a secondary chemotactic effect. Most interesting, we show that the migration is specific to undifferentiated NPCs and is not observed in the differentiated progeny of NPCs. Finally, we demonstrate that EGF signaling plays a role in the speed of the migratory behaviour with little effect on the directedness. We suggest that harnessing the migratory potential of NPCs in the presence of an electric field *in vivo* may provide means to enhance endogenous neurorepair and tissue regeneration elicited by SE-derived NPCs.

## Materials and Methods

### Ethics Statement

All animal work was approved by the University of Toronto Animal Care Committee in accordance with the institutional guidelines (protocol no. 20008754).

### Cell Culture

NPCs were obtained as previously described [19]. Briefly, adult CD1 mice were sacrificed by cervical dislocation. Brains were dissected, and the periventricular region was enzymatically dissociated. Cells were plated at 10 cells/µL in T25 or T75 culture flasks (BD Falcon, Canada) in SFM (DMEM∶F12 3∶1) supplemented with EGF (20 ng/mL; Sigma-Aldrich, Canada), basic fibroblast growth factor (bFGF, 10 ng/mL; Sigma-Aldrich, Canada) and heparin (2 µg/mL; Sigma-Aldrich, Canada) [20], [21]. After 7 days, primary neurospheres formed consisting purely of nestin-positive NPCs [20]. Primary neurospheres were either utilized for migration analysis or passaged and replated for another 7 days to form secondary neurospheres before being plated into galvanotaxis chambers.

### Galvanotaxis chamber construction

Galvanotaxis chamber construction was adapted from Zhao et al. [22]. Briefly, galvanotaxis chambers were constructed by sealing acid-washed square no. 1 glass cover slides (22 mm×22 mm×0.17 mm) (VWR, Canada) to the base of 60×15 mm plastic Petri dishes with silicon vacuum grease (VWR, Canada). A pair of rectangular glass slide pieces (22 mm×5 mm×0.17 mm) were sealed to opposite edges of the square slide to yield a central chamber with dimensions 22 mm×10 mm×0.17 mm (Figure S1). The chambers were then UV sterilized for 15 minutes. The central troughs were coated with 100 µg/mL poly-L-lysine (Sigma-Aldrich, Canada) for 2 hours at room temperature, rinsed 3 times with 1 mL of autoclaved water, and then incubated in 4% (v/v) Matrigel (BD Biosciences, Canada) in SFM for 1 hour at 37°C. The troughs were rinsed twice with SFM and covered with 300 µL of SFM supplemented with either EGF, bFGF and heparin, or fetal bovine serum (FBS, Invitrogen-Gibco, Canada) - depending on the experiment being performed - until ready to be plated with neurospheres. In experiments investigating the galvanotactic properties of undifferentiated NPCs, primary or first passaged neurospheres were plated on the chamber for 17–20 hours in the presence of EGF, FGF, and heparin at 37°C, 5% CO_2_ and 100% humidity. In contrast, neurospheres were plated into chambers for 69–72 hours in the presence of 1% FBS in SFM for experiments investigating the galvanotactic properties of NPCs induced to differentiate into mature phenotypes.

### Galvanotaxis assay

Neurospheres plated into the chamber were covered with a square no. 1 glass cover slide to create a roof to the central trough, yielding a central chamber. A media reservoir was created on either side of the chamber and sealed with vacuum grease so as to permit electric current flow only through the chamber. Two 15 cm length PVC tubes (Fisher Scientific, Canada) (2.38 mm inner diameter, 3.97 mm outer diameter) were filled with 1.5% agarose gel. Silver wire (Alfa Aesar, USA) was cut into two 10 cm pieces, coiled and immersed in Javex bleach for 20 minutes to form Ag/AgCl electrodes. The galvanotaxis chamber to be analyzed was placed onto the stage of a Carl Zeiss Axiovert 200 M microscope (Zeiss, Germany) that was situated within a temperature- and CO_2_-controlled, 100% humidity encasing. Each Ag/AgCl electrode was placed in a 60×15 mm Petri dish that was filled with 7.5 mL of SFM. These Petri dishes were placed on the microscope stage on either side of the Petri dish containing the galvanotaxis chamber. The agarose gel tubes were used to bridge the Petri dishes in order to establish electrical continuity between all three dishes (Figure S1). The Ag/AgCl electrodes were connected to an external constant-voltage power supply to establish a dcEF of strength 250 mV/mm [16]–[17] across the galvanotaxis chamber in the direction of the positive X-axis. Cell migration was recorded via time-lapse imaging microscopy using Zeiss Axiovision software, with images being captured at a frequency of one per minute for 2.5–8 hours. Cells were viewed at 5× for the largest field of view.

For cross-perfusion experiments, a microfluidic channel with two reservoirs (μ-Slide I, Ibidi, Germany) was pre-treated identically to the galvanotaxis chambers described above. Neurospheres were plated into the chambers in SFM+EGF, bFGF and heparin, and incubated for 17–20 hours as previously described. Each reservoir was filled with 1 mL of SFM+EGF, bFGF and heparin, PTFE thread sealant tape was wrapped around the rim of each of the channel's reservoirs and the lids were replaced onto the reservoirs to create a tight seal. A 16G1^½^ stainless steel needle was inserted into each reservoir. The needles served two purposes: i) they were hollow and therefore permitted fresh media perfusion and ii) they were metallic and therefore electrically conductive. The chambers were secured to the microscope stage and a peristaltic pump (Ismatec, Switzerland) was connected to the inlet and outlet terminals of the chamber via the 16G1^½^ needles to perfuse fresh SFM+EGF, bFGF, and heparin at a flow rate of 0.83 mL/min. The electrodes of the external power supply were connected directly to the needles to form a dcEF of strength 250 mV/mm across the galvanotaxis chamber.

### Immunocytochemistry

Cells were fixed with 4% paraformaldehyde for 20 minutes at room temperature directly in the galvanotaxis chambers and then washed 3 times with PBS for 5 minutes each. Cells were permeabilized with 0.3% Triton X-100 for 20 minutes at room temperature, followed by a triple wash with PBS for 5 minutes each time. Blocking was performed with 10% NGS (Jackson Immunoresearch Laboratories, Canada) in PBS for 1 hour at room temperature. Cells were incubated overnight in primary antibody at 4°C. The following day the chambers were washed three times with PBS for 5 minutes each time, and incubated at 37°C for 1 hour with secondary antibody. Primary and secondary antibody incubations were repeated for all antigens of interest. The following primary and secondary antibodies were used: *primary:* mouse monoclonal anti-nestin (1∶400, Millipore, Canada), and rabbit polyclonal anti-GFAP (1∶500, Sigma, Canada); *secondary*: goat-anti-mouse conjugated with Alexafluor 568 (1∶400, Invitrogen-Gibco, Canada), and goat-anti-rabbit conjugated with Alexafluor 488 (1∶400, Invitrogen-Gibco, Canada). Nuclear staining was performed with mounting medium containing DAPI (Vector Laboratories, Canada). Samples were stored at −20°C until they were imaged.

### Quantification of cell migration

Cell migration was tracked via Zeiss Axiovision software's automated tracking module. In order to ensure that cells could be followed for the duration of tracking, cells were selected for kinematic analysis if they were at least one cell body away from the nearest cell thereby decreasing the likelihood of cells overlapping each other during migration. For cells that were closer than one cell body to the surrounding cells manual tracking was performed using Zeiss Axiovision's tracking module. Cell position was determined by cell centroid locations. A minimum of 45 cells from at least 3 separate experiments were analyzed for each experimental group. Four kinematic parameters were analyzed.

1. *Displacement* in the direction of the positive X-axis (which is parallel to the direction of the dcEF vector when dcEF is applied) was analyzed over 2.5 hours, because this was the maximum time common to all experimental groups that the dcEF state remained constant (i.e., the maximum time before the dcEF was either reversed or eliminated).
2. The magnitude of velocity (herein referred to as *velocity*) was defined as the total displacement between the initial and final positions of the cells divided by total experimental time.
3. *Directedness* was obtained by dividing the displacement along the dcEF vector by the total (x,y)-displacement between the initial and final positions of the cells. Directedness was taken as positive in the direction of the dcEF (positive X-axis) and negative in the direction opposite the dcEF (negative X-axis).
4. *Tortuosity* was defined as the total path length of the cells' migration divided by the displacement between the initial and final cell positions.

The latter two parameters (3 and 4) characterize the extent to which the cells migrate in a straight line toward the cathode; a value of 1 for both directedness and tortuosity indicate a perfect straight-line migration parallel to, and in the direction of, the positive X-axis. In experiments where the direction of the dcEF was reversed, cells were deemed to have switched direction once their centroid exhibited a displacement in the direction of the new cathode for a minimum of two consecutive frames.

### Statistical analysis

All values are presented as group means ± S.E.M. Unless otherwise specified, differences between group means were determined using one-way ANOVA. Two-way ANOVA analysis with Bonferroni post-hoc tests were performed to determine if there was an interaction effect between the state of the dcEF and the differentiation state of the cells on the migratory behaviour of the cells. In all cases, statistical significance was set at *p*<0.05.

## Results

### Undifferentiated NPCs undergo cathodally-directed galvanotaxis in the presence of a dcEF

**Figure 1:**
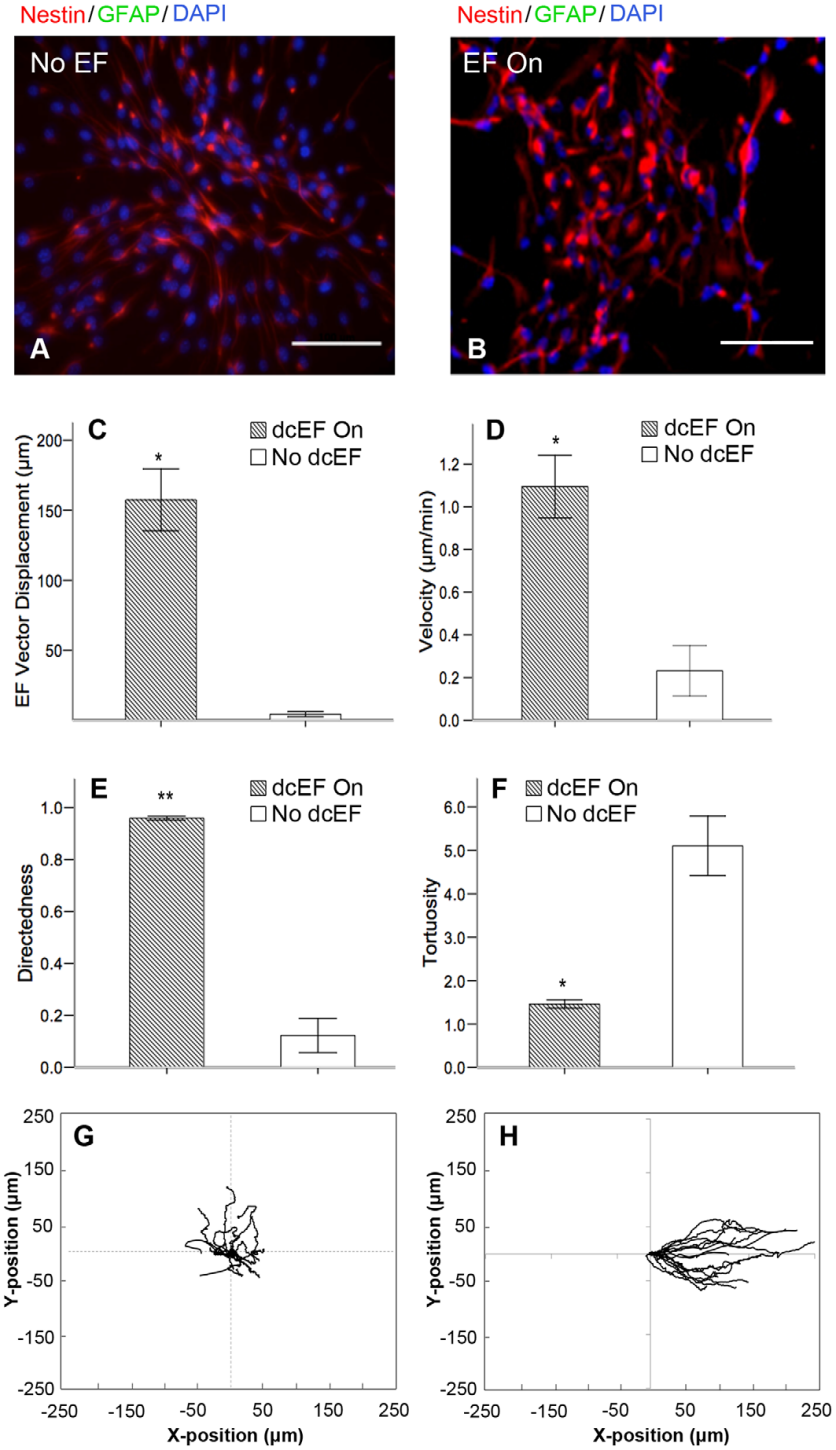
Undifferentiated NPCs undergo rapid and cathodally-directed migration in the presence of a dcEF. (**A–B**) NPCs are positive for nestin after 17 h on matrigel in the presence of EGF, bFGF and heparin (**A**) as well as after 6 h of dcEF exposure in the presence of EGF, bFGF and heparin (**B**). (**C–F**) NPCs exposed to a dcEF (n = 4) exhibit a larger dcEF-vector displacement (**C**), larger velocity (**D**), larger directedness (**E**), and smaller tortuosity (**F**), compared to NPCs not exposed to a dcEF (n = 3). (**G–H**) Typical cell migration paths for NPCs either not exposed (**G**), or exposed (**H**), to a dcEF of strength 250 mV/mm. Scale bars = 100 um. Data are presented as means ± S.E.M., * = *p*<0.01, ** = *p*<0.001.

We determined the pattern of migration that the NPCs exhibit in the presence or absence of an applied external dcEF. Neurospheres were plated into Matrigel-coated galvanotaxis chambers in growth factor conditions (EFG, FGF and heparin) to maintain the NPCs in an undifferentiated state for the duration of plating and imaging. Immunocytochemical analysis revealed the presence of nestin^+^ cells, verifying that the cells remained undifferentiated following 20 hours of culture on the chambers (Figure 1A). Neurosphere cells that adhered and dissociated in the chambers were analyzed for migration using time-lapse imaging for a period of 2.5–8 hours in either the absence or presence of a dcEF (250 mV/mm). Post-imaging immunostaining analysis revealed nestin expression was maintained in the NPCs regardless of the presence or absence of a dcEF when maintained under growth factor conditions (Figure 1A and 1B).

In the absence of a dcEF, mean cell body displacement over 2.5 hours in the direction of the cathode was 5.72±1.63 µm vs. 157.35±22.11 µm in the presence of a dcEF (Figure 1C and Figure S2). In the absence of a dcEF cells migrated in a random, non-directed manner at a mean velocity of 0.23±0.12 µm/min, a mean directedness (defined as the total dcEF vector displacement divided by the straight-line distance between initial and final cell positions) of 0.12±0.07, and mean tortuosity (the total path length of cell migration divided by the straight-line distance between initial and final cell positions) of 5.10±0.69 away from the central core of the dissociated neurosphere (Figure 1D–1G, Movie S1). Striking was the observation that when exposed to a dcEF, these undifferentiated NPCs underwent cathodal galvanotaxis at a rate of 1.09±0.15 µm/min, >4 times that seen in the absence of a dcEF (p<0.05). The directedness increased to 0.96±0.01 (a 9-fold increase, p<0.05), and tortuosity was reduced >3-fold to 1.56±0.10 (p<0.05), compared to precursors not exposed to a dcEF (Figure 1D, 1E, 1H, and Movie S2). Hence, undifferentiated NPCs exposed to a dcEF undergo rapid and cathodally-directed galvanotaxis, whereas their migratory behaviour in the absence of a dcEF is slower and non-directed. During exposure to a dcEF the NPCs were observed extending filopodia toward the cathode (Movie S3).

To ensure that the rapid and directed migration observed in the presence of the dcEF was a property of the majority of NPCs, and not due to their relative position within the cell cluster, we analyzed the migration of all cells within the field of view for a subset of three experiments using the manual tracking module of Zeiss Axiovision software. We found that 98.9%±0.4% of cells (1147 cells analyzed) had a positive overall displacement in the direction of the dcEF, indicating that galvanotactic migration is not a feature of a smaller sub-population of NPCs, but rather a phenomenon observed at the population level. Intraexperimental analyses between single cells that satisfied the cell selection criteria (cell situated near the outer edge of the dissociated neurosphere and at least one cell body away from the nearest cell) compared with cells that were situated near the cores of dissociated neurospheres revealed a significant difference in the velocity of migration (1.22±0.10 µm/min vs. 0.56±0.04 µm/min respectively, *p*<0.05) and displacement along the dcEF vector (176.69±15.16 µm vs. 77.71±5.87 µm respectively, *p*<0.05), but no change in tortuosity (1.37±0.05 vs. 1.32±0.03) or directedness (0.97±0.01 vs. 0.92±0.02). Hence, cells in higher density regions (at the centre of the clusters) migrated with a lower velocity than cells that were situated in lower density regions (near the perimeter of the cluster). Since cells can serve as physical obstructions to the migration of other cells, the regions of lower cell density may be more permissive to galvanotaxis than higher-density regions. For this reason, we focused the remainder of our analyses on cells residing near the outer edge of the dissociated neurosphere.

**Figure 2:**
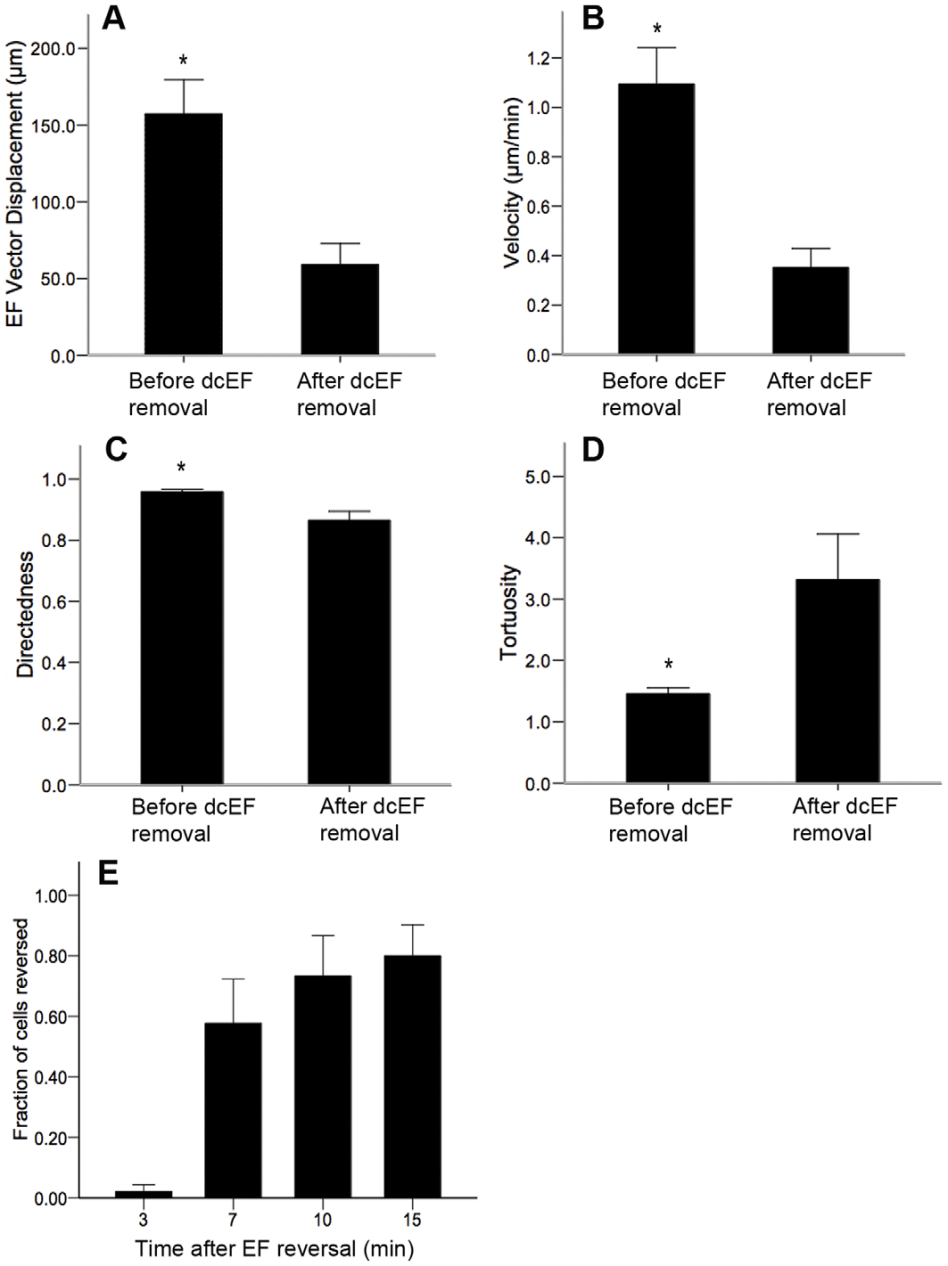
NPC galvanotaxis persists only for as long as the dcEF stimulus is present. (**A–D**) Analysis of cell migration for 20 minutes before and 20 minutes after the removal of the dcEF reveals a significant decrease in dcEF vector displacement (**A**), velocity (**B**), and directedness (**C**) of migration, as well as a significant increase in tortuosity (**D**). Following the reversal of the dcEF's direction, 80.0%±10.1% of cells analyzed reverse their direction of migration to point toward the new cathode within 15 minutes (**E**). Data are presented as means ± S.E.M (n = 3),* = *p*<0.005, ** = *p*<0.05.

To determine whether exposure to the dcEF had long-term effects on the behaviour of the NPCs, we examined the migratory behaviour immediately following the removal of the dcEF. We found that within 20 minutes following the abrupt removal of the dcEF, the NPCs reverted to a non-directed, random migration similar to that observed when the cells had not been exposed to a dcEF at all (Figure 2A–2D, Movie S4), and eventually their migratory behaviour became indistinguishable from NPCs that had never been exposed to a dcEF. Finally, we performed NPC galvanotaxis assays in which the direction of the dcEF was reversed after 2.5 hours so that it pointed in the direction of the negative X-axis instead of the positive X-axis. Strikingly, the majority of cells (80.0%±10.1%, 45 cells analyzed) reversed their direction of migration toward the new cathode within 15 minutes, although migration reversal was observed as early as 3 minutes (Figure 2E). Moreover, this reversal was observed at the population level as all cells within the field of view switched direction within 30 minutes. The cells maintained rapid and directed migratory behaviour toward the new cathode (Movie S5). Our results indicate that the cellular machinery required for NPC galvanotaxis can be actuated to induce migration – as well as reorganized to reverse migration – within 15 minutes of the dcEF onset or reversal, suggesting that de novo protein synthesis is not necessary for the process.

### Cells with differentiated neural phenotypes do not exhibit galvanotactic migration

**Figure 3:**
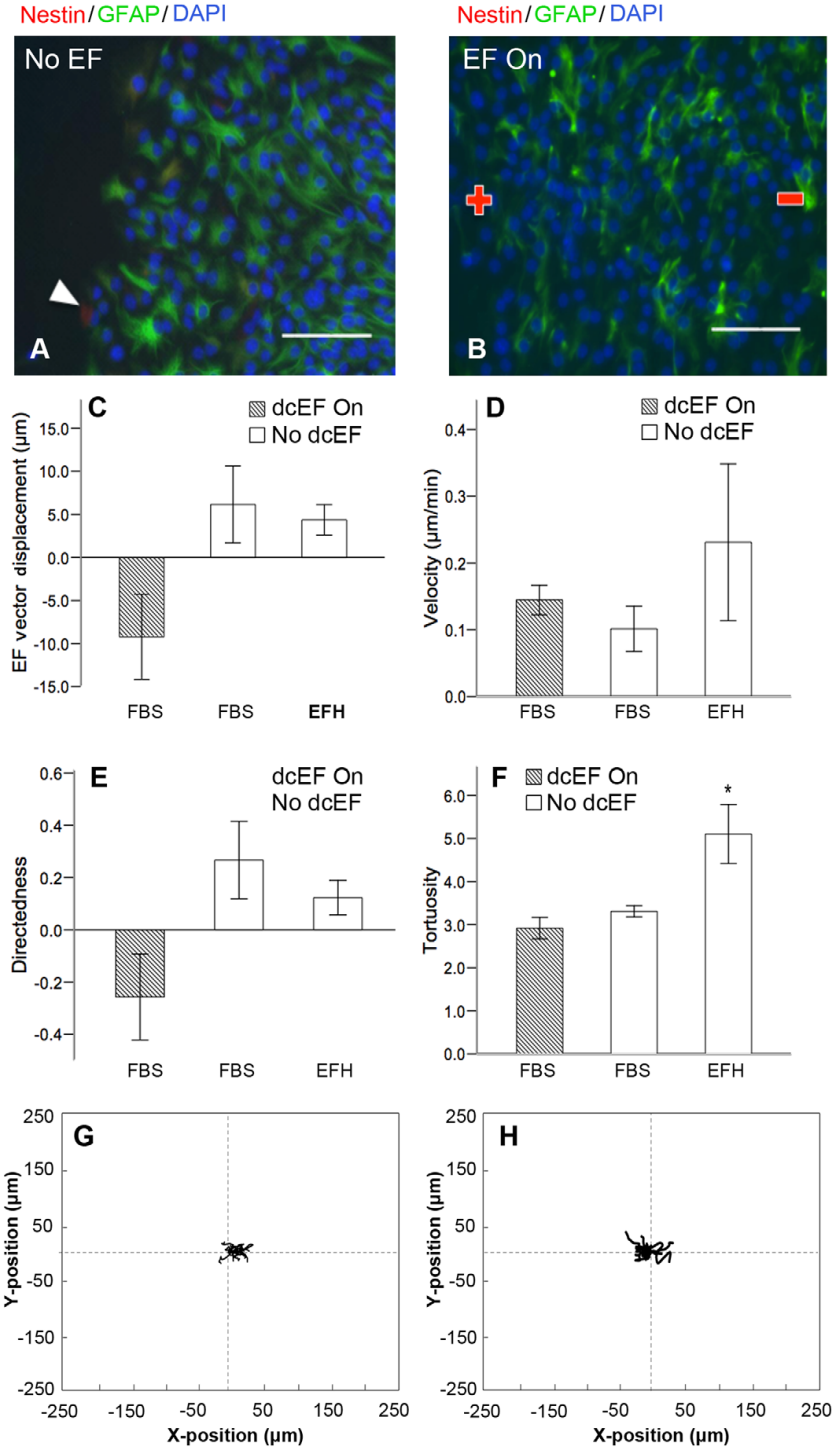
Differentiated NPCs do not exhibit directed migration in response to a dcEF. (**A–B**) cells are positive for the astrocytic marker GFAP after 69 h on matrigel in the presence of 1% FBS (**A**), as well as after 6 h of dcEF exposure in the same conditions (**B**). (**C–F**) Differentiated cells (FBS, dark bars, n = 4) exhibit no significant differences in dcEF-axis displacement (**C**), velocity (**D**), or directedness (**E**) when compared to differentiated cells in the absence of a dcEF (FBS, white bars, n = 3) and undifferentiated NPCs (EFH, white bars, n = 3) not exposed to a dcEF. However, undifferentiated NPCs in the absence of a dcEF exhibit greater tortuosity of migration than differentiated neural cells (**F**). (**G–H**) Typical cell migration paths for differentiated cells either not exposed (**G**), or exposed (**H**), to a dcEF of strength 250 mV/mm. Scale bars = 100 µm. Data are presented as means ± S.E.M, * = p<0.05.

We next asked if galvanotaxis is specific to undifferentiated NPCs or also a property of differentiated phenotypes. Neurospheres were plated into galvanotaxis chambers as described in the presence of 1% FBS for 69–72 hours to induce cell differentiation into mature neural phenotypes. Immunocytochemical analysis demonstrated that the majority of the cells expressed glial fibrillary acidic protein (GFAP) after 69 hours (Figure 3A), confirming that the NPCs had differentiated into astrocytes. This phenotype was maintained in FBS-cultured cells after 6 hours of dcEF exposure (Figure 3B). A rare subpopulation of cells continued to express nestin (Figure 3A, arrowhead) representing undifferentiated precursor cells. Differentiated cells were maintained in 1% FBS and either exposed or not exposed to a dcEF. Pre-differentiated cells exposed to a dcEF exhibited a mean −8.83±5.08 µm displacement in the direction of the dcEF–vector at a mean velocity of 0.14±0.02 µm/min (Figure 3C, 3D, S3, and Movie S6) Their directedness of migration was −0.26±0.16, with a mean tortuosity value of 2.92±0.25 (Figure 3E and 3F). This migratory behaviour did not differ significantly from that of differentiated cells that were not exposed to a dcEF or from undifferentiated cells in the absence of a dcEF (Figure 3C–3H, and Movie S7). Notably, post-dcEF labeling revealed that the differentiated cells (primarily astrocytes) aligned their processes perpendicular to the direction of the dcEF (Figure 3B).

**Figure 4:**
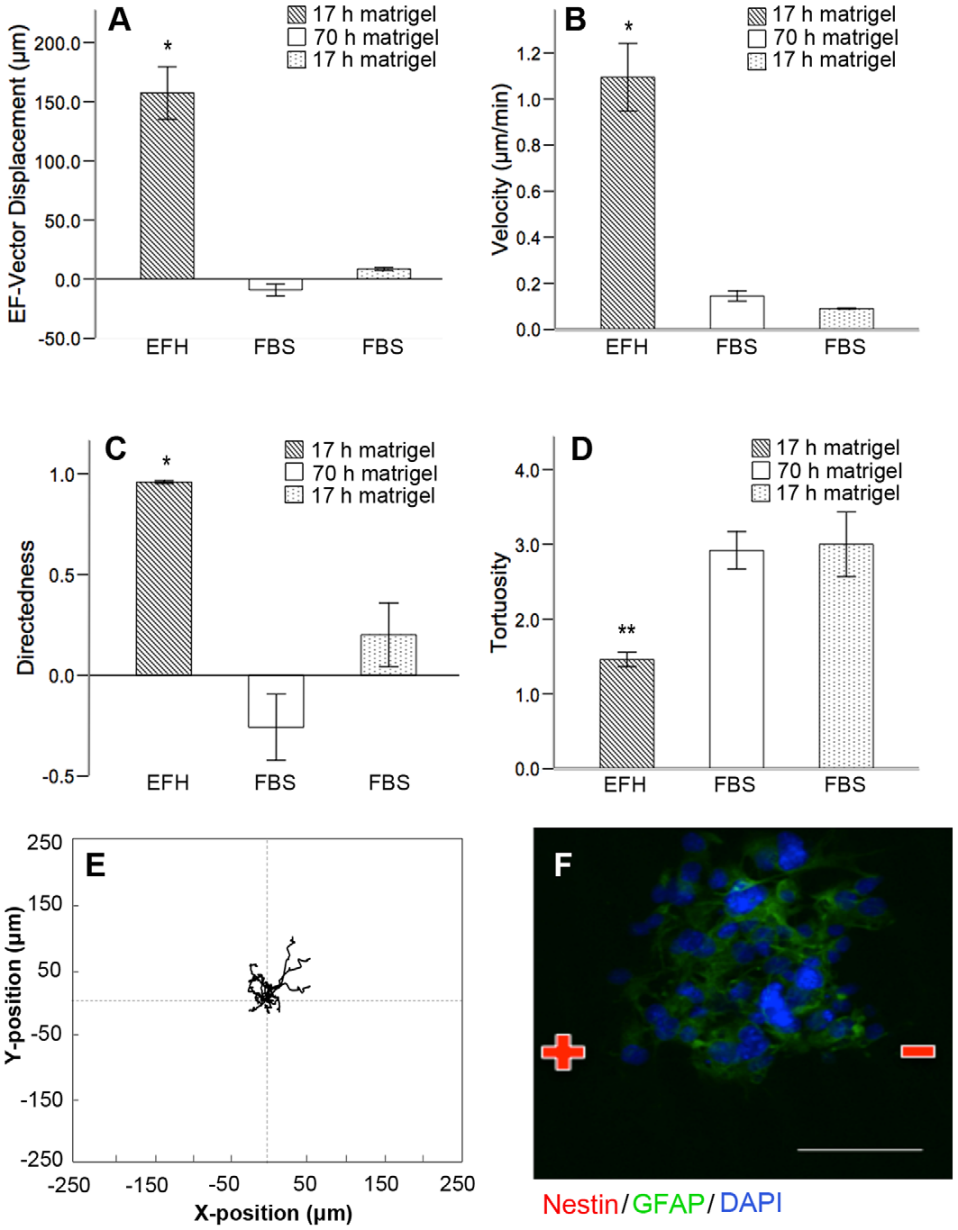
Prolonged adherence to Matrigel is not responsible for the loss of galvanotactic behaviour in differentiated cells. (**A–D**) Differentiated cells plated on Matrigel for 70 h (white bar, n = 4) or 17 h (dotted bars, n = 3) exhibit similar dcEF-axis displacement (**A**), velocity (**B**), directedness (**C**), and tortuosity (**D**). Allowing the differentiated cells to adhere to Matrigel for an equal amount of time as the undifferentiated cells (hatched bar, n = 4) does not prevent the loss of galvanotactic behaviour (**A–D**). (**E**) Typical cell migration paths for cells cultured in the presence of FBS for 70 hours and plated on Matrigel for 17 hours. (**F**) Immunostaining post dcEF-application reveals that the majority of cells are GFAP^+^ indicating that the NPCs have differentiated after 72 hours in FBS and 20 hours on Matrigel. Scale bar = 50 µm. Data are presented as means ± S.E.M, * = p<0.001, ** = p<0.005.

We considered that the lack of galvanotactic behaviour observed among differentiated cells could be due to the prolonged period of time that the cells are adhered to the Matrigel substrate in the galvanotaxis chambers prior to dcEF exposure. We asked if differentiated cells would undergo galvanotaxis if they adhered to the Matrigel substrate for only 17 hours, similar to the length of time that undifferentiated NPCs were maintained and exhibited galvanotaxis. Accordingly, NPCs were cultured for 52 hours in 1% FBS as free-floating neurospheres, and subsequently plated into Matrigel-coated galvanotaxis chambers for 17 hours in 1% FBS prior to application of the dcEF. We observed no significant difference in the migratory behaviour of differentiated cells after 17 hours versus ∼70 hours of adhesion (Figure 4A–4E) indicating that the lack of galvanotactic behaviour is due to their differentiated state, and not the prolonged binding period to Matrigel that is required to achieve FBS-induced maturation. Immunostaining post-dcEF application verified that the cells had differentiated (Figure 4F).

**Figure 5:**
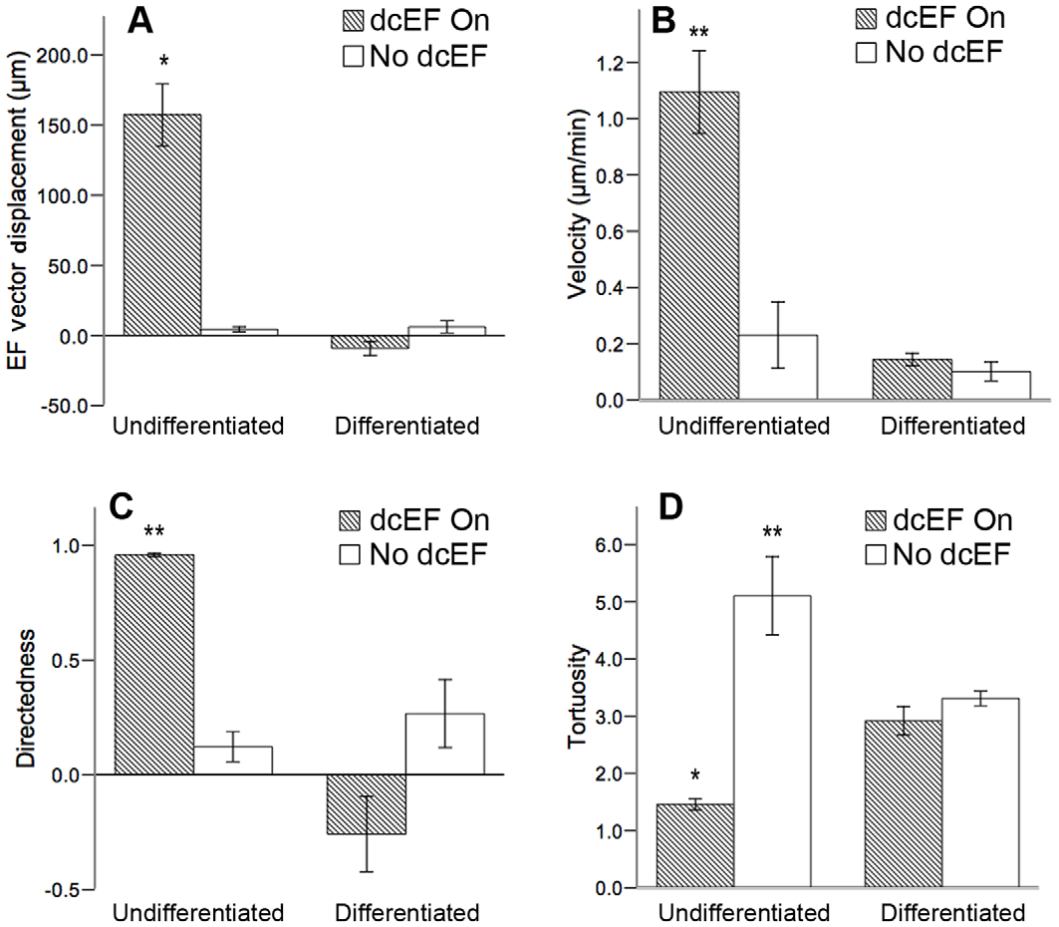
Summary of migratory properties of undifferentiated NPCs and differentiated cells in the absence and presence of a dcEF. (**A–D**) Undifferentiated NPCs exposed to a dcEF (n = 4) exhibit larger dcEF-axis displacements (**A**), larger velocities (**B**), larger directedness (**C**) and smaller tortuosities (**D**), compared to NPCs in the absence of a dcEF (n = 3), as well as compared to their differentiated counterparts in both the absence (n = 3) and presence (n = 4) of a dcEF. Data are presented as means ± S.E.M, * = p<0.001, ** = p<0.05.

Studies have indicated that the galvanotactic response of corneal epithelial cells [23], keratinocytes [24], and hippocampal precursors [17] is dependent on EGF receptor (EGFR) signaling. We asked whether the lack of galvanotaxis exhibited by differentiated cells (in FBS conditions) was due to the lack of exogenous growth factors in the culture media during exposure to the dcEF. NPCs were first exposed to differentiation conditions (FBS) for 69–72 hours on matrigel-coated chambers. Following this, the culture medium was aspirated and replaced with growth factor-supplemented medium (as is used to maintain NPCs in a precursor state) and the pre-differentiated cells were then immediately exposed to a dcEF. We found that growth factor-supplemented media failed to rescue galvanotaxis in cells that had been pre-cultured in differentiation conditions for 69–72 hours (Figure S4). Notably, differentiated cells transferred to growth factor conditions exhibited similar velocity and tortuosity compared to differentiated cells maintained in FBS conditions at all times. Interestingly, although differentiated cells consistently displayed low displacement in the direction of the dcEF and low directedness of migration, differentiated cells transferred to growth factor conditions showed a tendency to increased displacement towards the cathode relative to differentiated cells maintained in FBS at all times. Taken together, this suggests that the lack of rapid and cathodally-directed migration in differentiated cells is not due to the lack of EGF and bFGF signaling in the cells, and that growth factor signaling may impact the direction, but not the velocity, of these cells' migration. Figure 5 summarizes and compares the migratory behaviour of both differentiated and undifferentiated cells in either the absence or presence of a dcEF using 2-way Anova analysis.

### Electrically-induced NPC migration is a direct effect of the electric field

The conductivity of the culture media is imparted by its electrolyte constituents. As such, the existence of charged molecules within the media render the possibility of an electric field-induced redistribution of the electrolytes to form a chemotactic gradient [25]–[27]. We asked whether the observed directed migration of the NPCs was a direct effect of the dcEF, or if the cells were responding to a dcEF-induced chemical gradient. To eliminate the possibility of a chemical gradient forming within the galvanotaxis chamber, we designed a novel chamber that permitted the continuous perfusion of fresh SFM+EGF, bFGF, and heparin. The dcEF was maintained in the direction of the positive X-axis as in previous experiments, while media was continuously perfused in the direction of the negative X-axis, opposing the electric current flow.

**Figure 6:**
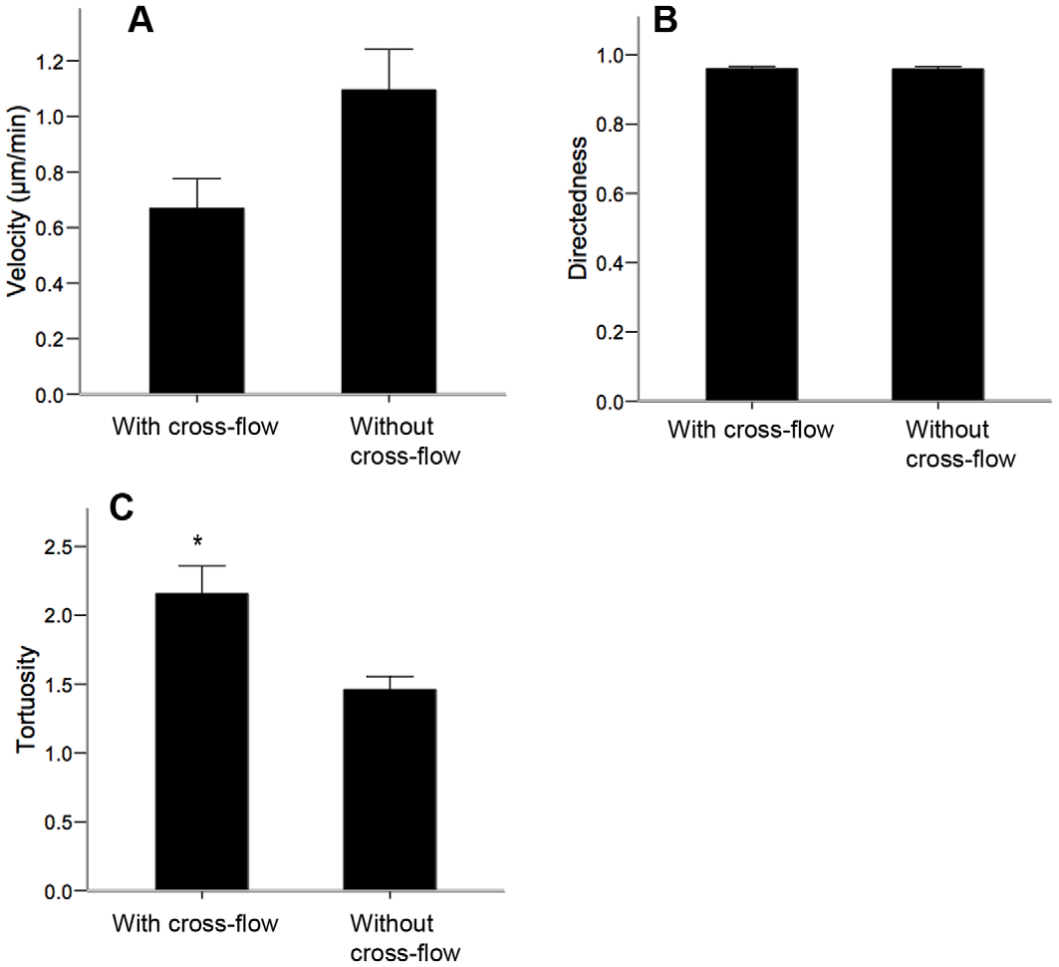
The galvanotactic behavior of undifferentiated NPCs is a direct effect of the applied dcEF. (**A–C**) NPCs exhibit no significant differences in the velocity (**A**), and directedness (**B**), of galvanotaxis when in either the presence (n = 4) or absence (n = 3) of a continuous cross-flow of media although they exhibit a higher tortuosity (**C**). Data are presented as means ± S.E.M, * = p<0.05.

Remarkably, the NPCs migrated in a directed manner against the shear stress of the fluid flow toward the cathode, albeit with a higher tortuosity than that of NPCs in the presence of a dcEF without SFM cross-perfusion (2.16±0.20 vs. 1.56±0.10) (Figure 6A–6C, Movie S8). There were no statistically significant differences in the velocity and directedness of migration between these two groups. Hence, these results demonstrate that the directed nature of the NPCs' migration in the presence of a dcEF is indeed a galvanotactic effect, and not a chemotactic effect induced by the dcEF.

### The galvanotactic response of undifferentiated NPCs to an externally applied dcEF involves EGFR signalling

**Figure 7:**
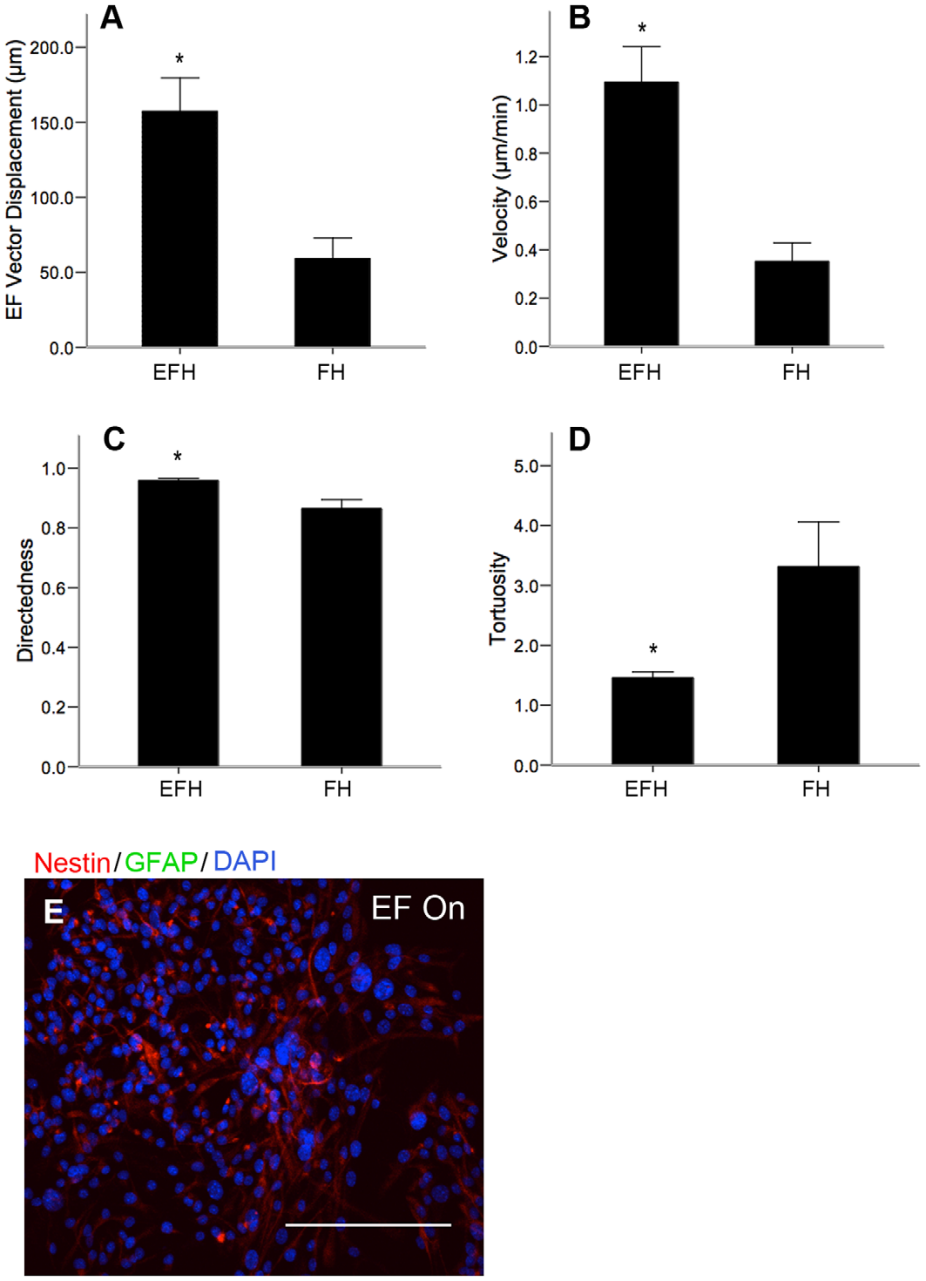
EGF is involved in regulating the rapid and directed NPC migration when exposed to a dcEF. (**A–D**) NPCs that are only exposed to bFGF and heparin (FH, n = 3) during dcEF application undergo smaller dcEF-axis displacements (**A**), lower velocity (**B**), lower directedness (**C**), and higher tortuosity (**D**), of migration compared to NPCs maintained in the presence of EGF, bFGF and heparin (EFH) at all times (n = 4). (**E**) NPCs remain positive for nestin following dcEF exposure in the presence of FH only. Scale bar = 100 µm. Data are presented as means ± S.E.M, * = p<0.05.

**Figure 8:**
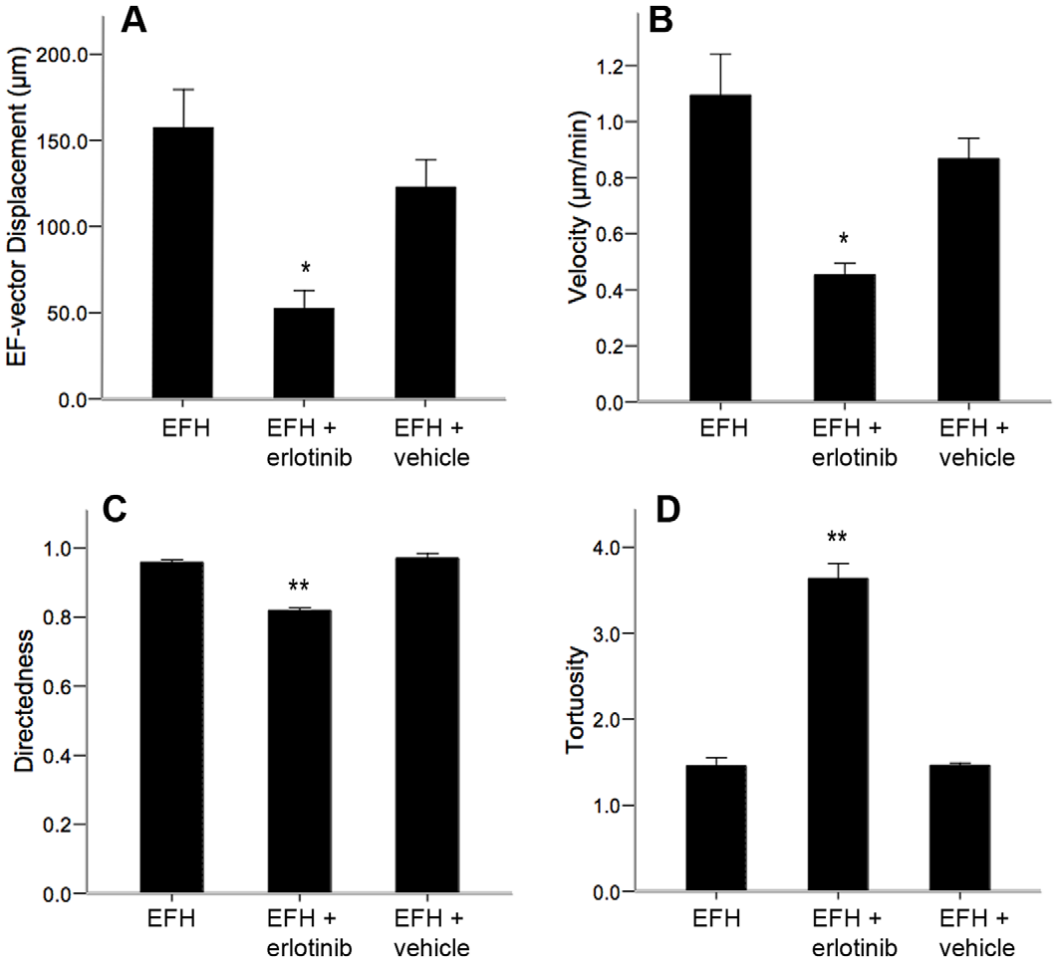
Epidermal growth factor signaling plays a role in the galvanotactic response of NPCs. (**A–D**) NPCs exposed to a dcEF in the presence of the EGFR blocker erlotinib (n = 3) experience significantly reduced dcEF-axis displacement (**A**), velocity (**B**), and directedness (**C**), of migration, as well as significantly greater tortuosity (**D**), compared to NPCs in the absence of erlotinib (EFH, n = 4, and EFH+vehicle, n = 3). Data are presented as means ± S.E.M, * = p<0.05, ** = p<0.001.

We demonstrated that the lack of migration of differentiated cells was not due to the lack of EGF since the addition of EGF could not rescue the galvanotactic response of differentiated cells. Next we asked if EGF signaling was important for the migratory behaviour of undifferentiated SE NPCs as previously described for other cell types [17]. We plated undifferentiated neurospheres into galvanotaxis chambers as before for 17 hours. Following this, the media was aspirated from the chambers, the troughs were gently washed and fresh SFM supplemented only with bFGF and heparin was immediately applied into the chamber and media reservoirs. The bFGF was present in order to maintain the NPCs in an undifferentiated state. Time-lapse imaging revealed that in the absence of EGF, NPCs exhibited significantly reduced dcEF-axis displacement (62.4%±18.7% reduction), velocity (67.8%±17.7% reduction), and directedness (9.8%±3.2% reduction) of migration, as well as significantly increased tortuosity (112.9%±48.7% gain) (Figure 7A–7D) compared to NPCs maintained in the presence of EGF at all times. We further demonstrated a role for EGF signaling in NPC galvanotaxis using the EGFR blocker, erlotinib which inhibits EGFR tyrosine kinase activity by preventing EGFR autophosphorylation via competitive binding to the ATP binding domain [28]. NPCs cultured in the presence of growth factors (EGF, FGF2 and heparin), with erlotinib (5 µg/mL in dimethyl sulfoxide, Santa Cruz Biotechnology, USA) migrated at a significantly decreased velocity, and increased tortuosity relative to vehicle controls and NPCs in growth factor conditions alone (without erlotinib) (Figure 8A–8D, Movie S9). Immunostaining verified that the NPCs remained nestin-positive after 2.5 hours of dcEF exposure in the presence of erlotinib, suggesting that FGF2 is sufficient to maintain cells in an undifferentiated state within this time period (Figure S5). Notably, even in the absence of EGFR signaling (no EGF, or the presence of erlotinib) NPCs maintained their directional bias toward the cathode, with only a 14% reduction in directedness, relative to cells in the presence of EGFR signaling. This suggests that EGFR signaling predominantly impacts the velocity – and to a lesser extent the directedness – of SE NPC galvanotaxis. Further, the migratory behaviour of NPCs exposed to a dcEF in the absence of EGF was not significantly different from that of NPCs in the presence of erlotinib. Taken together, these data suggest that while EGF signaling plays a role in the galvanotactic response of NPCs, it is not responsible for all the cell behaviours observed.

## Discussion

We have demonstrated that clonally-derived pure populations of adult SE-derived NPCs exhibit rapid and directed galvanotaxis toward the cathode of a dcEF. Moreover, we have shown this phenomenon to be unique to undifferentiated NPCs; inducing their maturation into differentiated phenotypes is associated with a loss of electrically-induced migratory capacity. Through continuous fresh media cross-perfusion experiments, we show that directed migration of NPCs in the presence of an applied dcEF is a direct effect of the field rather than an indirect chemotactic effect. We have provided evidence that NPC galvanotaxis is moderated by EGF signaling; both the removal of EGF from the culture medium as well as the blockade of EGFR via erlotinib significantly attenuate NPC galvanotaxis. Most interesting is the finding that loss of galvanotactic behaviour associated with FBS-induced maturation of NPCs could not be reversed by replacing the cells in the presence of EGF and bFGF. Thus our data indicate externally applied dcEFs can stimulate and guide the migration of undifferentiated SE NPCs, but not that of NPCs induced to differentiate into mature neural phenotypes.

The role of electric fields in the central nervous system has been previously explored. The axons of embryonic rat hippocampal neurons align perpendicular to the direction of an applied dcEF in an EF strength-dependent manner after 24 hours of exposure [29], and interestingly, individual growth cones of dendrites, but not axons, undergo cathodal orientation [30]. Xenopus embryo neural tube cells have been shown to elicit EF strength-dependent cathodal turning of neurites, although the direction of neurite growth in response to an applied dcEF varies depending on the substrate adhesiveness and net surface charge; negatively charged substrates such as laminin promote cathodal outgrowth, whereas positively charged substrates such as lysine promote anodal outgrowth [31], [32], reviewed in [33]. dcEFs also serve to modulate neuronal structure through differential neurite growth rate regulation (anode-facing neurites exhibit significantly slower outgrowth rates compared to cathode-facing neurites), and by enhancing neurite branching (predominantly cathodally) [12], [33], [34]. Interestingly, electric field exposure has been reported to impact the differentiation profile of NPCs. In higher strength dcEFs (437 mV/mm) adult rat hippocampal NPCs exhibit a tendency to differentiate into neurons, whereas the differentiation profile of embryonic mouse NPCs encapsulated in alginate hydrogel beads and exposed to lower-strength (1–16 mV/mm) alternating current EFs is dependent on the frequency and duration of stimulation [35], [36]. While these studies investigated the neurite response or differentiation of relatively stationary somata in the presence of a dcEF, we were interested in the entire cell body translocation of NPCs.

The findings reported here are similar to those of a recent study by Meng et al. [17], in which they showed that NPCs derived from an adult rat hippocampal cell line, as well as embryonic rat NPCs, undergo enhanced speed and cathodal directedness of migration in the presence of a dcEF. We have extended these findings to contrast and compare the galvanotactic capacity of undifferentiated SE NPCs with differentiated neural phenotypes. We have quantified total cellular displacement in the direction of the cathode as well as the tortuosity of migration (total path length divided by total displacement). The directedness measure of an individual cell's migration can vary based on the initial and final time points chosen for analysis. The mean tortuosity, in combination with mean directedness, is a more informative indicator of how straight the cells migrate toward a particular direction than directedness alone. The SE-derived NPCs exhibited markedly higher velocity of migration, as well as increased directedness, compared to the hippocampal NPCs described by Meng et al. [17] in the presence of the same dcEF strength (250 mV/mm) and growth factor conditions. This may suggest that electrical stimulation of adult NPCs with a dcEF may yield differential migratory responses depending on the region of the brain from which the cells originate, although we cannot rule out the possibility that these observed differences are due to the differing substrates utilized in each study (poly-L-lysine/matrigel vs. polyornithine/laminin). A recent study demonstrated the galvanotaxis of postnatal rat hippocampal neurons [37] suggesting that maturing cell phenotypes can also respond to EFs during times of active neurogenesis. To our knowledge, the galvanotaxis of adult-derived mature neural cell types has not been shown. With the long-term goal of developing endogenous neurorepair paradigms, our findings that differentiated neural cells do not exhibit a galvanotactic response suggest that dcEF application may be a suitable approach to the development of such paradigms.

The cellular mechanisms involved in NPC migration have not yet been fully characterized. EGFR signaling has previously been shown to play a role in the galvanotaxis of several cell types including keratinocytes [24], breast cancer cells [38], corneal epithelial cells [23], and embryonic NPCs [17]. It has been suggested that EGFR polarization within the membrane leads to actin colocalization and polymerization, and these processes in turn trigger cathodal galvanotaxis [22]. Indeed the activation and polarization of EGFR toward the cathode-facing side of NPCs was demonstrated in [17]. EGF has been extensively used to study NPC proliferation both *in vitro* and *in vivo* [19], [20], [39]. Phosphoinositide 3-OH kinase (PI3K) is a well-known downstream effector of the EGFR [40]–[41]. Rho GTPases (Rac1, Cdc42) are downstream targets of PI3K products and play key roles in the cytoskeleton remodeling process that is required for cell migration [42]–[43]. Meng et al. demonstrated that pharmacological and genetic inhibition of PI3K signaling significantly attenuated embryonic and hippocampal adult NPC migration [17]. Here we demonstrate that EGF also plays a role in the galvanotaxis of SE-derived NPCs. In the presence of the EGFR inhibitor erlotinib, undifferentiated NPCs experience significantly reduced migratory behaviour in the presence of a dcEF. However, their galvanotactic response is not completely eradicated in the presence of erlotinib, suggesting EGF is not exclusively responsible for NPC galvanotaxis. This is in line with the finding of Meng et al. [17] that FGF receptors are also involved in NPC galvanotaxis. In contrast to their findings, however, SE-derived NPCs in the presence of bFGF alone exhibited a significant decrease in the velocity and directedness of migration compared to NPCs in the presence of both EGF and bFGF. This suggests that the mechanisms by which growth factors mediate galvanotaxis may vary between hippocampal and SE-derived NPCs.

The identification of neural stem cells in the adult brain has led to the development of endogenous neural precursor activation paradigms to repair the injured CNS [3], [44]–[45]. Critical to the success of such self-repair paradigms is the effective expansion and recruitment of NPCs to sites of injury or disease. Although SE NPC expansion occurs following injury alone, or in combination with exogenous factors, only a subpopulation of the newly formed NPCs migrate toward lesion sites in response to these stimulants [46]. Augmentation of neurorepair processes may be achieved by enhancing the numbers of SE-derived NPCs that are recruited to lesion sites, and our findings suggest that this may be accomplished with the application of external dcEFs as guidance cues for NPC migration.

## Supporting Information

Figure S1 **Illustration of galvanotaxis chamber setup for time-lapse imaging.** The central Petri dish contains the galvanotaxis chamber, in which the cells are plated. The media inside the Petri dish housing the galvanotaxis chamber is supplemented either with EGF, bFGF and heparin, or with 1% FBS. The Petri dishes on either side of the galvanotaxis chamber are filled with SFM, and also contain the Ag/AgCl electrodes. These electrodes are connected to an external power supply. The three Petri dishes are connected in series with agarose-gel bridges.
(TIF)

Figure S2 **Undifferentiated NPCs undergo cathodal galvanotaxis.** (**A–D**) Time-lapse images of undifferentiated NPCs in the presence (**A,B**) or absence (**C,D**) of a 250 mV/mm dcEF at 0 (**A,C**) and 300 (**B,D**) minutes.
(TIF)

Figure S3 **NPCs that are induced to differentiate into mature phenotypes do not undergo cathodal galvanotaxis.** (**A–D**) Time-lapse images of differentiated neural cells in the presence (**A,B**) or absence (**C,D**) of a 250 mV/mm dcEF at 0 (**A,C**) and 300 (**B,D**) minutes.
(TIF)

Figure S4 **Growth factor conditions fail to rescue differentiated neural cell galvanotaxis.** (**A–D**) NPCs induced to differentiate into mature phenotypes and then plated back into growth factor conditions exhibit low displacement in the direction of the dcEF (**A**), as well as low velocity (**B**), low directedness (**C**) and high tortuosity (**D**) of migration. This behaviour is similar to cells maintained in FBS conditions at all times, although cells transferred to growth factor conditions tend to display preferential overall displacement toward the cathode. * = **p**<0.05.
(TIF)

Figure S5 **NPCs remain undifferentiated following 2.5 hours in the absence of EGFR signalling.** (**A–B**) NPCs maintain nestin-expression after 2.5 hours of EGFR blockade with erlotinib, both in the absence (**A**), and presence (**B**) of a dcEF. Scale bar = 100 µm.
(TIF)

Movie S1 **Time-lapse video of undifferentiated NPCs migrating cathodally in the presence of a dcEF.** NPCs were plated into galvanotaxis chambers for 17 hours in the presence of EGF, bFGF and heparin, and then exposed to a dcEF of strength 250 mV/mm for 2.5–8 hours. 1 second of video = 15 minutes real time.
(MP4)

Movie S2 **Time-lapse video of undifferentiated NPCs migrating in a random, radial manner in the absence of a dcEF.** NPCs were plated into galvanotaxis chambers for 17 hours in the presence of EGF, bFGF and heparin, and then imaged for 2.5–8 hours. 1 second of video = 15 minutes of real time.
(MP4)

Movie S3 **Time-lapse video of undifferentiated NPCs extending lamellipodia in the presence of a dcEF and growth factors EGF, bFGF and heparin.1 second of video = 15 minutes of real time.**
(MP4)

Movie S4 **Time-lapse video of undifferentiated NPCs in the presence of growth factors reverting to random migratory behaviour after the abrupt removal of the dcEF. 1 second of video = 15 minutes of real time.**
(MP4)

Movie S5 **Time-lapse video of undifferentiated NPCs in the presence of growth factors reversing their direction of migration toward the new cathode after the abrupt reversal of the dcEF's direction. 1 second of video = 15 minutes of real time.**
(MP4)

Movie S6 **Time-lapse video of differentiated NPCs in the presence of a dcEF.** NPCs were plated into galvanotaxis chambers for 69–72 hours in the presence of FBS, and then exposed to a dcEF of strength 250 mV/mm for 2.5–8 hours. 1 second of video = 15 minutes real time.
(MP4)

Movie S7 **Time-lapse video of differentiated NPCs in the absence of a dcEF.** NPCs were plated into galvanotaxis chambers for 69–72 hours in the presence of FBS, and then imaged for 2.5–8 hours. 1 second of video = 15 minutes real time.
(MP4)

Movie S8 **NPC migration in the presence of a dcEF is not a chemotactic effect.** NPCs maintain cathodal migratory behaviour even in the presence of continuous media cross-perfusion in the direction opposite to the dcEF. NPCs were plated into galvanotaxis chambers for 17 hours in the presence of EGF, bFGF and heparin, and then exposed to a dcEF of strength 250 mV/mm for 2.5–8 hours with fresh media continuously cross-perfused in the opposite direction. 1 second of video = 15 minutes real time.
(MP4)

Movie S9 **NPC migration is mediated by EGFR. In the presence of the EGFR blocker erlotinib, undifferentiated NPCs exhibit attenuated cathodal migration when exposed to a dcEF.** NPCs were plated into galvanotaxis chambers for 17 hours in the presence of EGF, bFGF and heparin. Subsequently, erlotinib was added into the culture medium and the NPCs were exposed to a dcEF of strength 250 mV/mm for 2.5–8 hours. 1 second of video = 15 minutes real time.
(MP4)
