# Supplementary figures and images for: Adult Subependymal Neural Precursors, but Not Differentiated Cells, Undergo Rapid Cathodal Migration in the Presence of Direct Current Electric Fields
Source: PLoS One. 2011 Aug 31;6(8):e23808. doi: 10.1371/journal.pone.0023808 (PMC3166127; doi:10.1371/journal.pone.0023808)

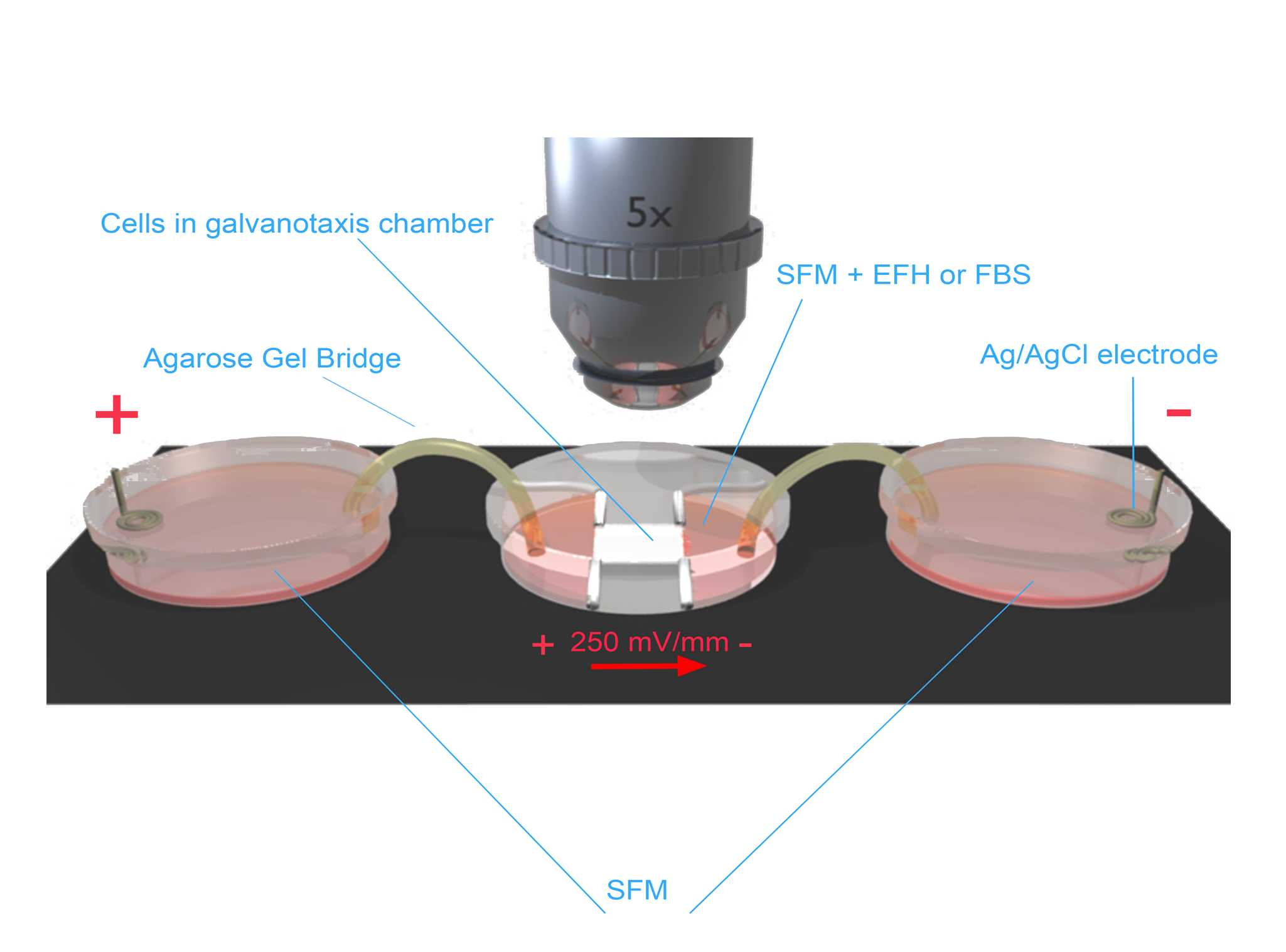

Supplement: Figure S1 — Illustration of galvanotaxis chamber setup for time-lapse imaging. The central Petri dish contains the galvanotaxis chamber, in which the cells are plated. The media inside the Petri dish housing the galvanotaxis chamber is supplemented either with EGF, bFGF and heparin, or with 1% FBS. The Petri dishes on either side of the galvanotaxis chamber are filled with SFM, and also contain the Ag/AgCl electrodes. These electrodes are connected to an external power supply. The three Petri dishes are connected in series with agarose-gel bridges. (TIF) [file pone.0023808.s001.tif]

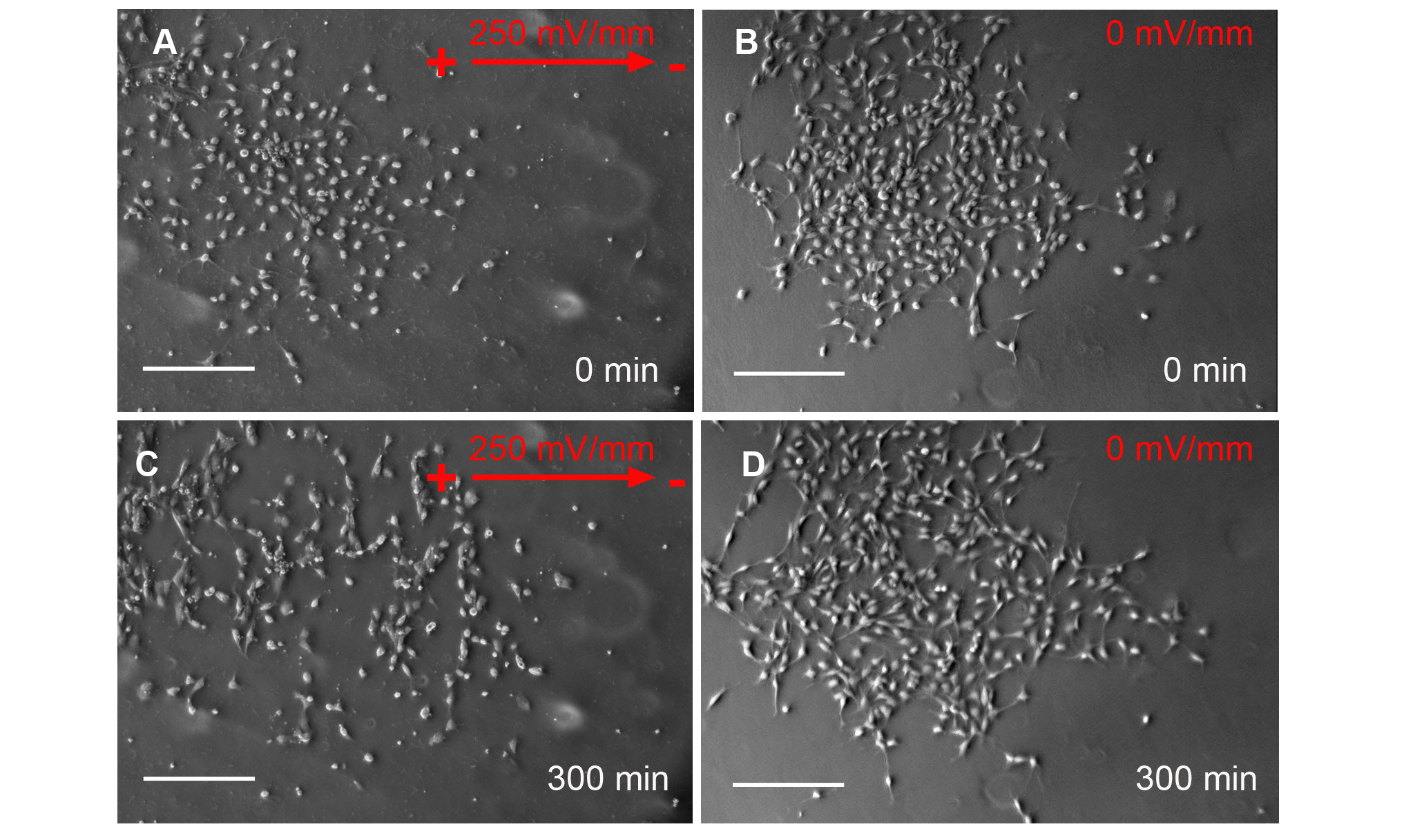

Supplement: Figure S2 — Undifferentiated NPCs undergo cathodal galvanotaxis. (A–D) Time-lapse images of undifferentiated NPCs in the presence (A,B) or absence (C,D) of a 250 mV/mm dcEF at 0 (A,C) and 300 (B,D) minutes. (TIF) [file pone.0023808.s002.tif]

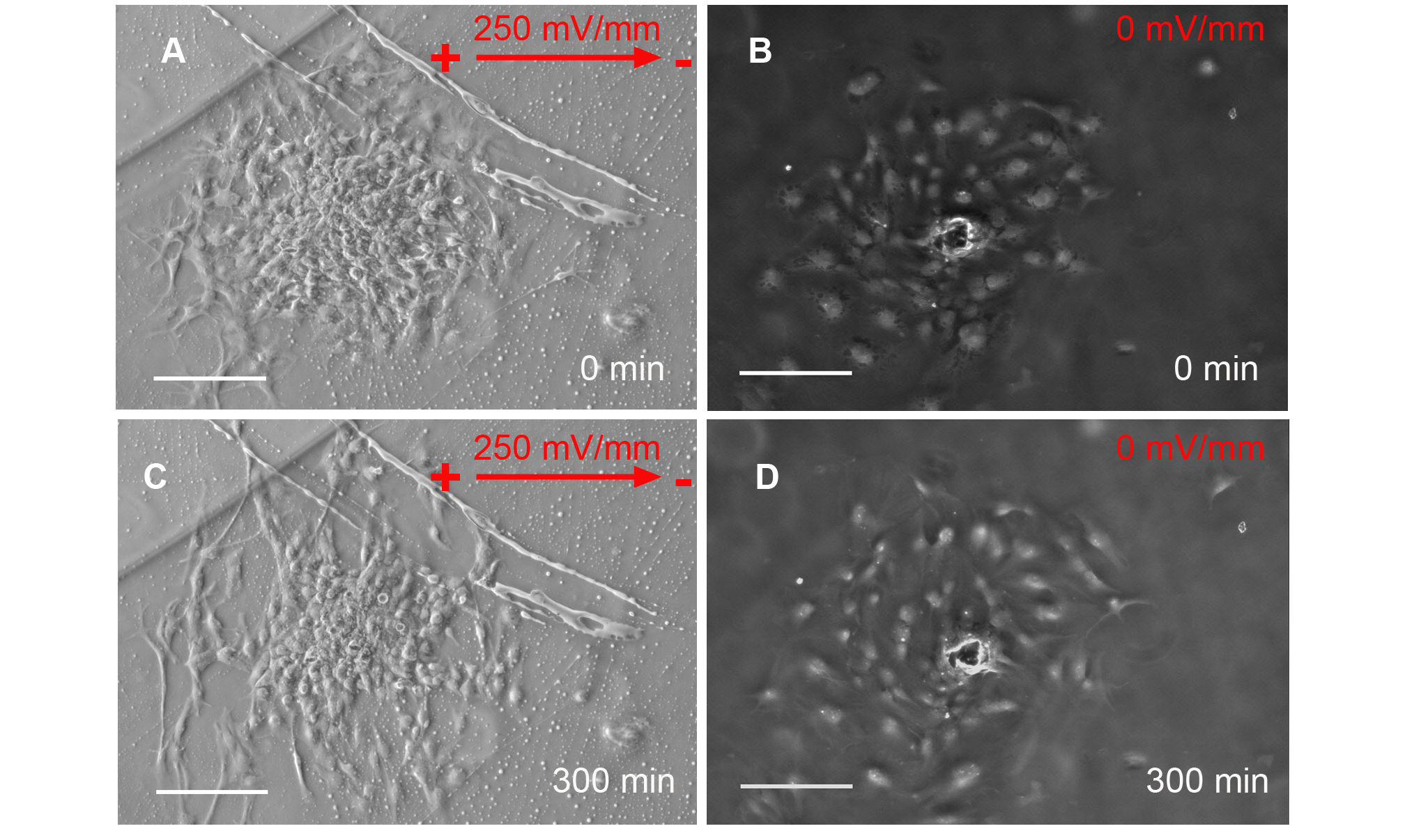

Supplement: Figure S3 — NPCs that are induced to differentiate into mature phenotypes do not undergo cathodal galvanotaxis. (A–D) Time-lapse images of differentiated neural cells in the presence (A,B) or absence (C,D) of a 250 mV/mm dcEF at 0 (A,C) and 300 (B,D) minutes. (TIF) [file pone.0023808.s003.tif]

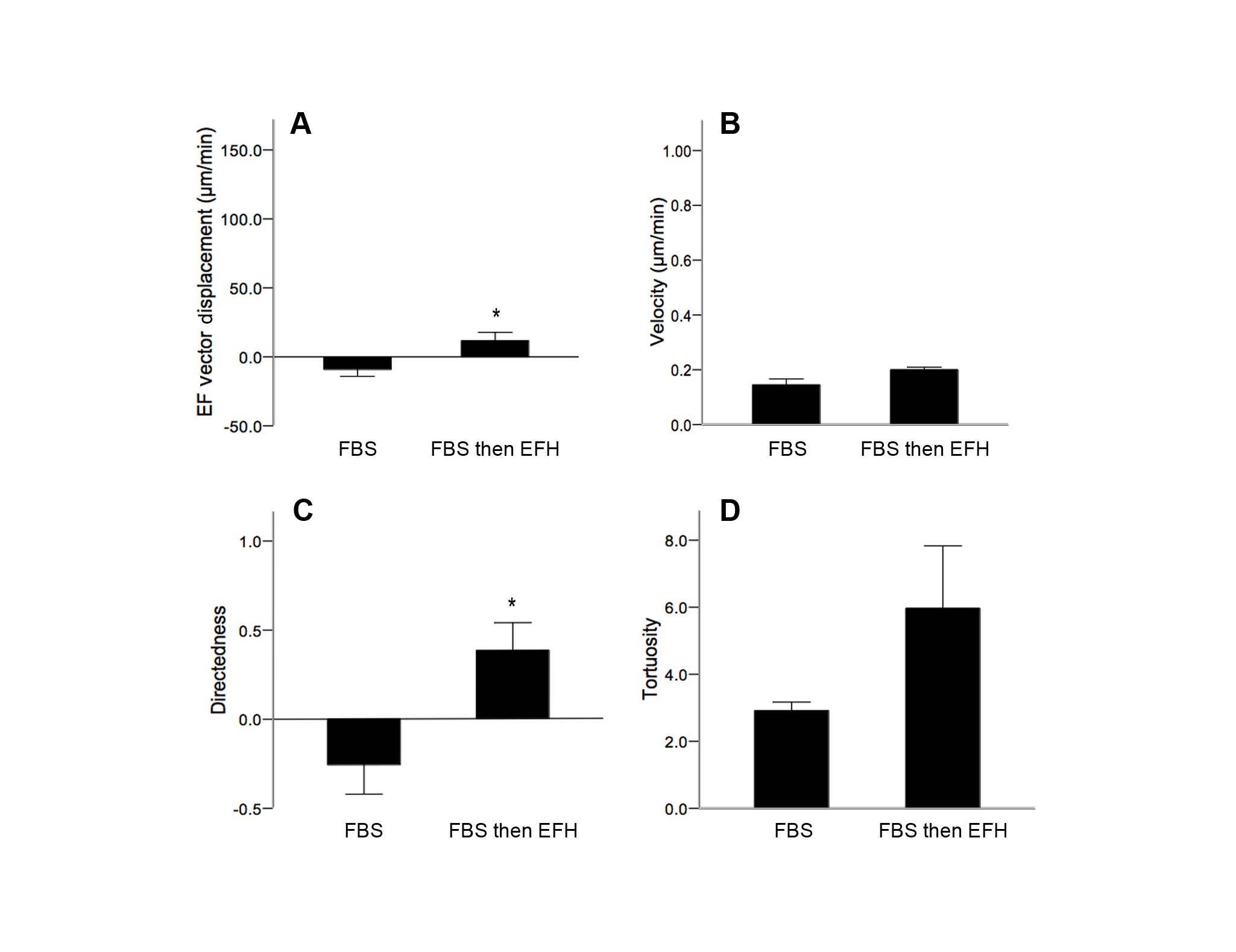

Supplement: Figure S4 — Growth factor conditions fail to rescue differentiated neural cell galvanotaxis. (A–D) NPCs induced to differentiate into mature phenotypes and then plated back into growth factor conditions exhibit low displacement in the direction of the dcEF (A), as well as low velocity (B), low directedness (C) and high tortuosity (D) of migration. This behaviour is similar to cells maintained in FBS conditions at all times, although cells transferred to growth factor conditions tend to display preferential overall displacement toward the cathode. * = p<0.05. (TIF) [file pone.0023808.s004.tif]

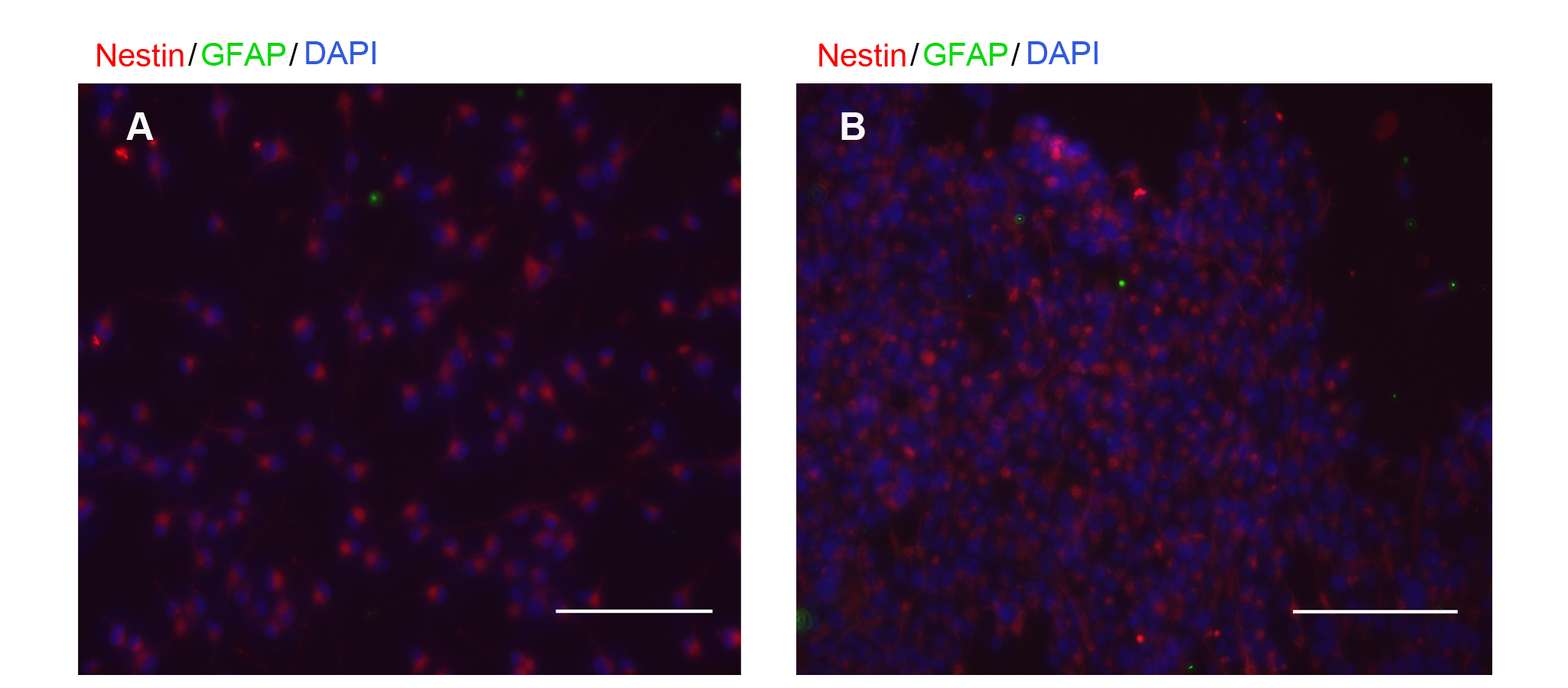

Supplement: Figure S5 — NPCs remain undifferentiated following 2.5 hours in the absence of EGFR signalling. (A–B) NPCs maintain nestin-expression after 2.5 hours of EGFR blockade with erlotinib, both in the absence (A), and presence (B) of a dcEF. Scale bar = 100 µm. (TIF) [file pone.0023808.s005.tif]
